# Affordance-Experimentation: Eine Fallstudie zur Entwicklung von Virtual-Reality-Anwendungsfällen im Unternehmenskontext

**DOI:** 10.1365/s40702-021-00828-7

**Published:** 2021-12-09

**Authors:** Jennifer Fromm, Elena Slawinski, Milad Mirbabaie

**Affiliations:** 1 Universität Duisburg-Essen, Duisburg-Essen, Deutschland; 2 Universität Paderborn, Paderborn, Deutschland

**Keywords:** Virtuelle Realität, Anwendungsfälle, Affordance, Experimentierphase, Fallstudie, Virtual Reality, Use Cases, Affordance, Experimentation Phase, Case Study

## Abstract

Durch technologische Fortschritte in den letzten Jahren ist Virtual Reality erschwinglicher und benutzerfreundlicher geworden, sodass Unternehmen die Einführung der Technologie verstärkt in Betracht ziehen. Ihren Aufschwung erlebte die Technologie jedoch durch die Unterhaltungs- und Spieleindustrie, weshalb sich für Unternehmen die Frage nach sinnvollen Anwendungsfällen stellt. Nach der Affordance-Experimentation-Actualization-Theorie ist insbesondere bei neu aufkommenden Technologien eine Experimentierphase notwendig, um Handlungsmöglichkeiten aufzudecken und daraus Anwendungsfälle zu generieren. Dieser Artikel präsentiert die Ergebnisse einer Fallstudie in einem Unternehmen, das sich während der Studie in der Experimentierphase befand. Durch Interviews mit acht Beschäftigten und einem Vertriebspartner konnten drei Handlungsmöglichkeiten für Virtual Reality im Unternehmenskontext und eine zuvor nicht bekannte Aktivität der Experimentierphase identifiziert werden. Damit erweitert die Studie bisherige Forschung zur Experimentierphase und zeigt Unterschiede im Vergleich zu anderen innovativen Technologien auf, die in vorherigen Studien untersucht wurden. Für Unternehmen bietet die Studie wertvolle Einblicke in die erfolgreiche Gestaltung der Experimentierphase als Vorbereitung auf die Implementierung.

## Abstract (en)

In recent years, technological advances have made virtual reality more affordable and user-friendly, encouraging companies to increasingly consider the adoption of this technology. However, the technology experienced its upswing through the entertainment and gaming industry, which is why companies face the challenge of generating meaningful use cases. According to the affordance-experimentation-actualization theory, an experimentation phase is necessary, especially for emerging technologies, to discover possible action potentials and generate use cases. This article presents the results of a case study in a company that has been in the experimentation phase during the study. Through interviews with eight employees and a sales partner, three action potentials for virtual reality in the corporate context and one previously unknown activity of the experimentation phase were identified. Thus, the study extends previous research on the experimentation phase and identifies differences compared to other innovative technologies examined in prior studies. For companies, the study offers valuable insights into how to successfully design the experimentation phase in preparation for the implementation of virtual reality.

## Einführung

Virtual Reality (VR) ermöglicht es in eine computergenerierte Welt einzutauchen und mit ihr zu interagieren. Die erfolgreiche Nutzung in Unternehmen blieb aufgrund hoher Kosten und geringer Qualität lange aus. Erst in den letzten Jahren erlebte VR besonders in der Unterhaltungs- und Spielebranche einen Aufschwung (Wohlgenannt et al. 2020). Die Erschwinglichkeit der Hardware und die steigende Qualität sorgten dafür, dass VR im Unternehmenskontext nun als eine vielversprechende Innovation gilt.

Ohne vorherige VR-Erlebnisse sind sinnvolle Anwendungsfälle für Unternehmen jedoch nicht ohne Weiteres zu erkennen, weshalb der Mehrwert noch mit Skepsis gesehen wird. In dieser Studie wird daher ein Unternehmen bei der Erforschung von Aktionspotenzialen und Generierung von sinnvollen Anwendungsfällen begleitet. Basis dafür bildet die Affordance-Experimentation-Actualization-Theorie (A-E-A-Theorie), die sich mit der Implementierung neuer Technologien beschäftigt (Du et al. 2019).

Unter Affordances versteht man Aktionspotenziale, die Technologien verschiedenen Akteuren bieten. Allerdings garantieren diese keine Ergebnisse, wenn sie nicht umgesetzt werden (Strong et al. 2014). Die effektive VR-Nutzung lässt sich somit als Prozess ansehen, in dem die Aktionspotenziale realisiert werden. Innovationen wie VR weisen meist keine etablierten Anwendungsfälle vor, daher muss eine Vorbereitung stattfinden, um Affordances wahrzunehmen und Anwendungsfälle zu generieren. Diesen Vorgang bezeichnen Du et al. (2019) als Experimentierphase. Diese Phase wurde erst in zwei Fallstudien zu Blockchain und Predictive Maintenance (PdM) untersucht, weshalb noch Forschung zur Übertragbarkeit der Aktivitäten auf VR vonnöten ist (Du et al. 2019; Keller et al. 2019). Daraus ergeben sich folgende Forschungsfragen:

1. Welche Affordances bietet VR für Unternehmen?
2. Wie können Unternehmen eine erfolgreiche VR-Experimentierphase gestalten?

Um diese Fragen zu beantworten, wurde eine Fallstudie in einem Unternehmen durchgeführt. Durch die Studie wurden drei VR-Affordances ermittelt. Zusätzlich ließen sich die Aktivitäten der Experimentierphase mit VR bestätigen und um die Aktivität der Testung ergänzen.

Die Studie erweitert die Forschung durch die Einbindung der Experimentierphase in ein Affordance-Prozess-Modell (Pozzi et al. 2014). Für Unternehmen gewährt die Studie einen Einblick in eine erfolgreiche VR-Experimentierphase.

## Konzeptuelle Grundlagen

### VR im Unternehmenskontext

Die VR-Technologie ermöglicht es Nutzenden, abgeschottet von der physischen Welt in eine virtuelle Umgebung einzutauchen (Wohlgenannt et al. 2020). Seit der Entwicklung in den 1960ern erlebte VR oftmals Aufschwünge, hatte aber auch mit Schwierigkeiten zu kämpfen (Fransson et al. 2020). Ein Meilenstein war im Jahr 2012 die Oculus Rift, mit der eine neue Generation von Head-Mounted Displays für den Konsumgütermarkt vorgestellt wurde. Ab 2016, mit der Entwicklung von bekannten Systemen, wie Playstation VR oder HTC Vive, ist VR für eine breite Masse zugänglich geworden. Das Jahr 2020 hat die Nutzung von innovativen Technologien durch die COVID-19-Pandemie weiter vorangetrieben.

VR bietet Organisationen diverse Aktionspotenziale, wie die nachhaltige Produktplanung bei Audi zeigt: Der Konzern demonstrierte ein Automodell, das mit 3D-Scans ausschließlich virtuell geplant und konstruiert wurde (Audi Media Center 2020). Audi hat damit eine effiziente und nachhaltige Methode der Produktplanung erreicht, die global durchgeführt werden kann. Forschende wiesen zudem auf die kreativitätsfördernde Wirkung in Design-Thinking-Prozessen hin (Fromm et al. 2020; Vogel et al. 2020). Darüber hinaus bietet VR neue Vermarktungsansätze von Produkt-Showrooms bis hin zu virtuellen Messeständen, die potenzielle Kundschaft näher an Produkte heranrücken lassen (Orsolits und Lackner 2020). Die realitätsnahe Demonstration von Funktionen sorgt für eine Abhebung gegenüber der Konkurrenz. Ebenso bietet VR einzigartige Möglichkeiten für erfahrungsbasiertes Lernen (Fromm et al. 2021) und eine Ergänzung zu traditionellen Schulungen: Die Deutsche Bahn bietet ihren Beschäftigten ein praxisnahes Training, um ICE-Züge betrachten zu können, die nicht dauerhaft für Schulungen bereitstehen (DB 2018).

### Affordance-Experimentation-Actualization-Theorie

Das Affordance-Konzept geht auf Gibson (1979) zurück, der untersuchte, wie Tiere die Aktionspotenziale ihrer natürlichen Umgebung wahrnehmen. Ebenso lassen sich auch Affordances von Technologien wie VR erforschen. So definierten Markus und Silver (2008) sie als *„possibilities for goal-oriented action afforded to specified user groups by technical objects.“* Auf einer abstrakten Ebene identifizierten Steffen et al. (2019) drei VR-Aktionspotenziale: negative Aspekte der physischen Welt vermindern (z. B. riskantes Training), existierende Aspekte der physischen Welt nachbilden (z. B. realistische 3D Modelle) und Aspekte schaffen, die in der physischen Welt nicht existieren (z. B. physikalische Gesetze umgehen). Einen Effekt erzielen Affordances jedoch erst, wenn sie in der Aktualisierungsphase realisiert werden (Strong et al. 2014). Der Prozess von der Existenz, über die Wahrnehmung bis hin zur Aktualisierung wurde in einem Affordance-Prozess-Modell zusammengefasst (Pozzi et al. 2014). Die A‑E-A-Theorie beinhaltet die Experimentierphase als zusätzliche Phase, die besonders bei innovativen Technologien von Bedeutung ist (Du et al. 2019).

In dieser Phase werden Anwendungsfälle durch drei Aktivitäten entwickelt, bevor ein Unternehmen zur Aktualisierung übergehen kann (Du et al. 2019). Bei der *konzeptionellen Erforschung* handelt sich um die Entwicklung eines grundlegenden Verständnisses der Technologie (Keller et al. 2019). Die Aktivität wurde in einer PdM-Fallstudie identifiziert, da ein Basiswissen erst durch Workshops angeeignet werden musste. Während der *konzeptionellen Anpassung* wird einer Technologie eine neue Bedeutung im Unternehmenskontext gegeben (Du et al. 2019). In einer Blockchain-Fallstudie wurde die Technologie mit Bitcoin verbunden. Die Entwicklung neuer Anwendungsfälle erforderte nun, sich davon zu lösen. Die *Einschränkungsminderung* umfasst die Erarbeitung von Lösungsvorschlägen für den Umgang mit potenziellen Einschränkungen (Du et al. 2019). In der Blockchain-Studie wurde u. a. die Komplexität der Technologie aufgeführt. Bei VR ist die Untersuchung der Experimentierphase relevant, da die Technologie durch ihren Ursprung oft mit Spielen assoziiert wird und sich für Unternehmen die Frage nach sinnvollen Einsatzmöglichkeiten stellt.

## Methode

Zur Beantwortung der Forschungsfragen wurde eine Fallstudie bei PureAir (Pseudonym) durchgeführt. PureAir wurde 1951 gegründet und konzentriert sich auf die Entwicklung, Herstellung und den Vertrieb von Lüftungssystemen. Es handelt sich um ein internationales Unternehmen mit ca. 4000 Beschäftigten und einem Umsatz von mehr als 500 Mio. €. Seit 2018 plant PureAir Einsatzmöglichkeiten für innovative Technologien im Unternehmen zu identifizieren. Im November 2019 wurde PureAir auf VR aufmerksam und startete ein Projekt in der internen Zukunftswerkstatt. Sechs Auszubildende erhielten die Aufgabe, erlebbare und wirtschaftliche VR-Anwendungsfälle zu identifizieren, wofür drei Softwarepakete von Drittanbietern (Tab. 1) herangezogen wurden. Anschließend wurden die Anwendungsfälle in einem Testlauf mit einem Vertriebspartner erprobt und das Unternehmen ging zur Implementierung über.

**Tab. 1:** Softwarepakete

Software	Ausgelegt für
WeAre	Erstellung virtueller Räume für Geschäftsmeetings
Simlab	Erstellung virtueller Räume für Produktausstellungen und Schulungen
Present4D	Erstellung virtueller Führungen

Es wurden Leitfaden-Interviews durchgeführt, basierend auf dem erworbenen Wissen durch erste Kontakte mit dem Unternehmen und Fragen aus bestehender Literatur. Die Teilnehmenden wurden zu den Projekt-Meilensteinen, verfolgten Zielen, VR-Einsatzmöglichkeiten und möglichen Einschränkungen befragt.

Insgesamt wurden acht Beschäftigte und ein externer Vertriebspartner von PureAir befragt (Tab. 2). Es handelt sich dabei um alle Beschäftigten, die bis zum Ende des Projekts besonders stark eingebunden waren, sowie den Vertriebspartner, mit dem der Testlauf durchgeführt wurde. Die Interviews wurden mit einer qualitativen Inhaltsanalyse ausgewertet (Mayring 2015). Es wurde ein deduktiv-induktiver Ansatz der Kategorienbildung verwendet, um sowohl bestehendes Wissen aufzugreifen als auch neue Erkenntnisse zu generieren. Deduktive Kategorien waren die drei Aktivitäten der Experimentierphase; induktiv wurden eine weitere Aktivität und drei Affordances identifiziert.

**Tab. 2:** Interview-Teilnehmende

ID	Position	Alter	Rolle im VR-Projekt
T1	Ausbildungsleiter IT	50	Gründer der Zukunftswerkstatt; Weitergabe der Aufgabenstellung zum VR-Projekt an Auszubildende
T2	Ausbildungsleiter Konstruktion	37	Gründer der Zukunftswerkstatt; Weitergabe der Aufgabenstellung zum VR-Projekt an Auszubildende
T3	Auszubildender IT	21	Teilnehmer der Zukunftswerkstatt; Entwicklung von Einsatzmöglichkeiten
T4	Bereichsleiter Brandschutz	45	Produktexperte und Präsentator der virtuell vorgestellten Brandschutzkomponenten
T5	Bereichsleiter IT	56	Mitglied des Tech Monitors; Weitergabe des VR-Themas an die Zukunftswerkstatt und die Ausbilder
T6	Bereichsleiterin Marketing	54	VR-Implementatorin im Bereich Marketing
T7	Bereichsleiter Vertrieb	45	Vermittelnde Instanz zwischen PureAir und Kunde
T8	Mitarbeiter Produktentwicklung für Filtertechnik	40	Produktexperte und Präsentator des virtuell vorgestellten Luftfilters
T9	Vorstandsmitglied eines Kunden (Vertriebspartner)	39	Externer Teilnehmer des Testlaufs

## VR-Einsatzmöglichkeiten

### Standortunabhängige Kollaboration

Die erste Affordance umfasst die Möglichkeit, inner- und außerbetrieblich standortunabhängige Kollaboration durchzuführen. Bei PureAir fand Zusammenarbeit bisher persönlich oder über digitale Plattformen statt. Eine Möglichkeit, um Geschäftsreisen und dazugehörige negative Aspekte zu reduzieren, ohne dass der persönliche Kontakt darunter leidet, bietet *WeAre* durch virtuelle Konferenzen:

Es können virtuelle Räume generiert werden, in welche die Teilnehmenden standortunabhängig mit einer VR-Brille eintauchen können. Sie können Besprechungen in einer realitätsnahen Umgebung führen und dabei in CAD (Computer-Aided Design) erstellte Produkte hineinladen und diskutieren. Ebenso kann *WeAre* interaktive Explosionszeichnungen hervorrufen: *„Das Modell explodiert, sagt man in Technik-Sprache, das heißt es wird in alle Einzelteile gezogen und man kann sich das Einzelteil rausgreifen, was nochmal interessant ist zu erklären“* (T8). Zusätzlich lassen sich Schnittebenen der Produkte darstellen, sodass das Innere des Produktes zum Vorschein kommt. T2 fügt hinzu, dass *„dieses Zerlegen selbst in CAD nicht so schnell möglich ist“*.

### Virtualisierung der Vermarktung

Zweitens ermöglicht VR die Vermarktung von Produkten zu virtualisieren. Bei Produktvorstellungen wurden bisher umfangreiche Informationsblätter verteilt oder der Vertrieb für die Vor-Ort-Präsentation herangezogen. Mit zunehmender Länge nahm das Engagement beim Lesen der Produktblätter deutlich ab. Zum anderen gibt es viele Produkte, die aufgrund der Größe oder des Gewichts nicht vor Ort vorgestellt werden können.

Da *WeAre* nur einen Raum anbietet, der nach jedem virtuellen Besuch zurückgesetzt wird, hat das Unternehmen für diese Zwecke eine weitere Software herangezogen. *Simlab* ermöglicht durch virtuelle Showrooms das standortunabhängige Präsentieren der Produkte in VR. Ebenso lassen sich die Produkteigenschaften und deren Funktionsweise im Detail betrachten „*und das natürlich in verschiedenen Größen*“ (T2). Selbst wenn die Standortunabhängigkeit außer Acht gelassen wird und „*wenn das Setup so ist, dass man vor Ort etwas aufbauen muss, ist es trotzdem extrem effektiv, weil nicht das ganze Produktportfolio durch die Gegend getragen werden muss*“ (T7).

*Present4D* ermöglicht eine virtuelle Führung über das Werksgelände als 360°-Video. T8 erklärt: „*Wir können das Werk hier einmal abfilmen und dann, sag ich mal à la Google Maps, so Standorte wählen, wo man was sieht und was zu sagen kann*.“

### Digitalisierung von Schulungen

Außerdem bietet VR die Möglichkeit, Schulungen zu digitalisieren: *„Wenn man eine Brandschutzklappe beispielsweise erklären möchte, dann hat man früher das Produkt auf den Tisch gestellt, alle drum herum versammelt und das erklärt“* (T6). Dabei war die Gruppengröße begrenzt, da sie *„sich gar nicht so nah positionieren können, sodass sie alles sehen“* (T4). Zudem mussten Maschinen dafür aus der Produktion genommen werden.

Hier lässt sich ebenso *WeAre* nutzen, womit sich das Innere von Produkten durch eine Explosion oder einen Schnitt betrachten lässt. Dies ermöglicht ein besseres Erklären der Produkte. Auch die limitierte Gruppengröße lässt sich aufheben, da physikalische Gesetze umgangen werden können und jeder an die Seite des Erklärenden gestellt werden kann. Mit der virtuellen Schulungsumgebung von *Simlab* können Produktions- oder Montageabläufe in einem sicheren Umfeld ohne reale Maschinen und Produkte erlernt werden. Am Beispiel einer Montage erklärt T2: „*Also die Bauteile liegen daneben, sogar ein Schraubendreher, und man nimmt dann Stück für Stück die Teile, positioniert sie und dreht sie auch fest. Das wäre die Anwendung, um einen Mitarbeiter einzuarbeiten.*“

## Gestaltung der Experimentierphase

### Konzeptionelle Erforschung

Einige Befragte konnten vor den betrieblichen Experimenten erste VR-Erfahrungen sammeln. Dennoch fand eine konzeptionelle Erforschung statt, da alle Teilnehmenden die neuen Softwarepakete kennenlernen mussten und die ersten Berührungspunkte häufig nicht im Unternehmenskontext stattfanden.

Zuerst wurde eine Einführung genutzt, die vom Vertreiber der Software im virtuellen Raum angeboten wurde: *„Da kommt dann einfach ein Kollege der Firma dazu und erklärt die Funktionen. Und dann springt er zum nächsten Kunden und erklärt es da in dem Raum“* (T2). So wurden die ersten Akteure im Umgang mit VR geschult und dazu befähigt, die VR-Softwarepakete eigenständig zu erkunden und deren Funktionen auszuprobieren. Durch die Anschaffung von ersten Testgeräten konnten die Teilnehmenden bspw. weniger involvierte Auszubildende oder Vertriebspartner ebenfalls in der Benutzung der VR-Hardware und -Software anleiten. Schlussendlich haben alle Teilnehmenden ein Basiswissen erworben.

### Konzeptionelle Anpassung

Die meisten Teilnehmenden haben VR in der Unterhaltungs- und Spielebranche kennengelernt, wodurch sich annehmen ließ, dass dies die Nutzung im Unternehmen beeinflussen könnte. Eine potenzielle Gefahr war, dass die Beschäftigten nur spielerische Anwendungen erkennen. Es stellte sich jedoch heraus, dass VR offen empfangen wurde. So erzählte T1: *„Da ich im Gaming-Bereich drin war und unsere Produkte alle neu für mich waren, habe ich am Anfang gesagt: ‚Hey, wäre doch total cool, wenn man unsere Produkte in so einem Raum hätte und da kann man dann drum herumlaufen und den Kopf reinstecken‘“*. Dieses Zitat demonstriert, dass die ersten Berührungspunkte in der Spielebranche die konzeptionelle Anpassung sogar erleichterten.

Außerdem wird VR zwar bereits von einzelnen Unternehmen eingesetzt, jedoch konnten deren Anwendungsfälle nicht ohne Anpassung auf PureAir übertragen werden. So erhielten die Teilnehmenden der Zukunftswerkstatt die Aufgabe, prototypische, aber skalierbare Anwendungsfälle zu entwickeln, die den Anforderungen des Unternehmens gerecht werden.

Zuerst wurden sämtliche Ideen gesammelt, die bei der ersten VR-Nutzung entstanden sind. Außerdem wurden bestehende Anwendungsfälle bei anderen Unternehmen gesammelt und überlegt, inwiefern diese für den lokalen Unternehmenskontext geeignet wären oder angepasst werden müssten. Anschließend recherchierten die Auszubildenden nach Softwareanbietern, die die Ideen umsetzen konnten. In diesem Zuge wurde bspw. diskutiert, ob VR für PureAir ähnlich wie bei Audi auch für die virtuelle Konstruktion von neuen Produkten unternehmensintern eingesetzt werden könnte. Jedoch wurde die Umsetzung als zu komplex für den Einstieg gesehen, da bestehende Softwarepakete ressourcenintensiv auf die speziellen Gegebenheiten bei PureAir angepasst werden müssten. Dementsprechend wurden eher Anwendungsfälle weiterverfolgt, die sich mit leichten Anpassungen an bestehenden Softwarepaketen umsetzen ließen. Nach und nach entstanden die auf PureAir zugeschnittenen Anwendungsfälle, die der Leitlinie *„Wenn ich nichts anpacken muss, muss ich auch nicht vor Ort sein“* (T2) folgten.

Die Zukunftswerkstatt war für die Experimentierphase von großer Bedeutung, da dort Auszubildende abseits des gewöhnlichen Arbeitsalltags frei mit der neuen Technologie experimentieren konnten. Es wurde dort nicht als Spielerei gesehen, wenn Auszubildende verschiedene Anwendungen ohne klare Zielsetzung testeten, um ein Verständnis für die Möglichkeiten von VR zu erlangen.

### Einschränkungsminderung

Bei PureAir wurden in der Zukunftswerkstatt ohne Unterbrechung des laufenden Betriebs erste Testversuche durchgeführt, in denen sich drei mögliche Einschränkungen herausstellten und Lösungswege erarbeitet wurden.

Der Besitz und die Installation der VR-Ausstattung wurden von acht Teilnehmenden als Einschränkung erwähnt. Es *„braucht natürlich jeder dieses Highend-Set auf der gegenüberliegenden Seite. Wenn das nicht da ist, funktioniert das ganze System nicht“* (T8). Diese Einschränkung wird besonders bei Produktpräsentationen vor Kunden deutlich, die nicht im Besitz der Technologie sind und eventuell auch nicht die Kosten dafür tragen möchten.

T2 schlug eine Lösung vor: *„Man kann in diesen Raum auch über den Rechner eintreten und hat dann wie beim Computerspiel eine Steuerung und kann sich einen Vortrag anhören. Aber das Erlebnis ist ein anderes*.“ Um Kunden teilhaben zu lassen, entstand die Idee eines VR-Sets, das den Kunden zugesandt werden könnte. Es wurde „*ein Köfferchen mit allem Drum und Dran [vorgeschlagen]. Schön beschrieben, dass man wirklich nur alles zusammenstecken braucht“ *(T1)*. *Das Set könnte außerdem in die PureAir-Roadshow eingebunden werden, bei der es sich um einen mobilen Präsentationsraum in einem PKW-Anhänger handelt, mit dem Beschäftigte im Vertrieb zur Kundschaft fahren, um Produkte persönlich vorzustellen. Mit VR wären nicht nur kleine Produkte sowie Informationsmaterialien vorzeigbar, sondern auch große Produkte und deren Einsatzmöglichkeiten.

Vier Teilnehmende äußerten, dass der menschliche Kontakt durch den Versand von VR-Sets in den Hintergrund rückt: „*Aus Vertriebssicht muss es ein gesunder Mix aus digital und persönlich sein*“ (T7). Zum Beispiel kann das VR-Erlebnis von Beschäftigten im Vertrieb begleitet werden: *„Man geht mit dem Koffer dahin. Vom Hauptsitz aus wird die Schulung gemacht und der Hauptansprechpartner ist den ganzen Tag mit den Leuten zusammen. Dann kommt die Nähe ja gar nicht zu kurz“* (T7). Außerdem muss es *„Kunden geben, die völlig unabhängig vom Event trotzdem persönlich besucht werden. Und das ohne VR-Brille, ohne alles. Hinsetzen, analoge Pizza essen, analoges Bier trinken und Spaß haben“* (T7).

Weitere drei Teilnehmende nannten die Simulatorkrankheit als Einschränkung. Es handelt sich hierbei um das Empfinden von Übelkeit aufgrund widersprüchlicher Wahrnehmungen des Seh- und Gleichgewichtssinns. T2 erzählte, dass auch eine Mitarbeiterin von PureAir betroffen war: *„Wir hatten eine Auszubildende, die konnte das nicht so gut vertragen. Ihr ist im Stehen schlecht geworden.“ *Diese Einschränkung stellt eine der größten Hürden in der VR-Nutzung dar, und Lösungsansätze wurden bisher nur mäßig benannt. T2 vermutete, dass *„das Schwindelgefühl abnehmen wird, wenn man mehr Zeit in der VR verbringt. Außerdem soll es besser werden desto höher die Qualität der VR ist.“*

### Testung

In der Fallstudie wurde die Testung als weitere, abschließende Aktivität der Experimentierphase identifiziert. So wurde das Projekt erst nach erfolgreichem Testlauf mit einem Großkunden als umsetzungsfähig eingestuft. Im Vergleich zur Zukunftswerkstatt, die Anwendungsfälle generieren sollte und die Technologie eher theoretisch betrachtet hat, war es das Ziel der Testung, die Arbeitsweise mit VR vor der internen Einbindung in einem realitätsnahen Fall zu erproben. Der Ablauf wurde wie folgt beschrieben: *„Wir sind dorthin gefahren. Wir haben dort diese VR-Station aufgebaut, haben den Bereich eingemessen, haben die Sensoren an den Ecken platziert. Im Hauptsitz waren zwei Kollegen, die zwei unterschiedliche Produkte erklärt haben. Das heißt, wir haben dort eine virtuelle Produktveranstaltung gemacht“ *(T7). Es wurde nicht nur der Showroom präsentiert, *„sondern auch die Kollaboration“ *(T1). T9 fügte noch hinzu: *„Ich bin positiv überrascht, auch wegen der Detailtreue. Und ich finde VR passt auch super zu technischen Produkten, ganz unabhängig von PureAir. Es ist eigentlich eine sehr gute Möglichkeit, erklärungsbedürftige Produkte zu zeigen.“* Nach positiver Resonanz wurde der Start der Implementierung eingeleitet, somit der Übergang zur Aktualisierungsphase.

## Diskussion und Fazit

In der Fallstudie konnten drei VR-Aktionspotenziale im Unternehmenskontext identifiziert werden: 1) standortunabhängige Kollaboration, 2) Virtualisierung der Vermarktung und 3) Digitalisierung von Schulungen. Des Weiteren bestand die Experimentierphase bei PureAir aus vier Aktivitäten: 1) konzeptionelle Erforschung, 2) konzeptionelle Anpassung, 3) Einschränkungsminderung und 4) Testung (Tab. 3).

**Tab. 3:** Übersicht zur Umsetzung der Aktivitäten in der Experimentierphase

Aktivität	Beschreibung	Maßnahme
Konzeptionelle Erforschung	Aktivität, in der konzeptionelles Verständnis der Technologie aufgebaut wird	Kennenlernen von VR-Hardware und -Softwarepaketen (Schulung durch Drittanbieter und eigenständiges Ausprobieren)
Konzeptionelle Anpassung	Aktivität, in der die Technologie vom ursprünglichen Kontext abgegrenzt wird und unternehmensspezifische Anwendungsfälle generiert werden	Abgrenzung von der Unterhaltungs- und Spielebranche, Recherche von bestehenden VR-Anwendungsfällen bei anderen Unternehmen und Anpassung auf den lokalen Unternehmenskontext innerhalb der Zukunftswerkstatt
Einschränkungs-minderung	Aktivität, in der Einschränkungen einer Technologie identifiziert und in einem weiteren Schritt gemindert werden	Aufbau von Hardware und Ausprobieren von Softwarepaketen zum Aufdecken von technischen, logistischen und organisatorischen Einschränkungen bei der Umsetzung der entwickelten Anwendungsfälle, Theoretische Überlegung von Lösungsansätzen
Testung	Aktivität, in der die unternehmensspezifischen Anwendungsfälle und Lösungsansätze zur Einschränkungsminderung getestet werden	Testen der entwickelten Anwendungsfälle und Lösungsansätze für Einschränkungen in realer Umgebung bei einem Großkunden, Evaluation des Feedbacks der internen und externen Testbeteiligten

### Einordnung der Ergebnisse

Die identifizierten Aktionspotenziale stehen im Einklang mit den übergeordneten VR-Affordances nach Steffen et al. (2019). In Bezug auf die Experimentierphase fanden Keller et al. (2019) heraus, dass nur wenige Teilnehmende einen grundsätzlichen Kenntnisstand über PdM vorwiesen und eine konzeptionelle Erforschung durchliefen, um Basiswissen zu erlangen. Dahingegen war VR vergleichsweise bekannt. Deshalb wurde die Aktivität angepasst und umfasst im VR-Kontext die Erforschung der neuartigen Software und Hardware.

Ähnlich wie Blockchain in einer Fallstudie mit Bitcoin assoziiert wurde (Du et al. 2019), waren bei VR vor allem spielerische Anwendungen bekannt. Dennoch gestaltete sich die konzeptionelle Anpassung nicht als problematisch. Die bekannten Anwendungsfälle aus der Spielebranche dienten sogar als Inspiration für die Entwicklung von sinnvollen Anwendungsfällen im Unternehmen.

Bei der Einschränkungsminderung lässt sich erkennen, dass obwohl VR lange existiert, deutlich mehr Einschränkungen erkannt wurden als bei Blockchain oder PdM (Du et al. 2019; Keller et al. 2019). Dies kann daran liegen, dass bei VR ein größeres Spektrum an Einsatzmöglichkeiten betrachtet wird. PdM gehört bspw. zur grundlegenden Technologie der künstlichen Intelligenz, sodass der Wartungsbereich nur einen kleinen Teil darstellt. Gleichzeitig hat sich VR in letzter Zeit stark entwickelt, weshalb voraussichtlich viele Einschränkungen im Laufe der Jahre gelöst werden. Die meisten ermittelten Einschränkungen stehen im Einklang mit Forschung von Fransson et al. (2020), die u. a. die hohen Kosten und die Simulatorkrankheit als Hürde nannten.

Im Vergleich zu bisherigen Studien (Du et al. 2019; Keller et al. 2019) wurde die Testung als weitere Aktivität der Experimentierphase identifiziert. Wichtig ist die Abgrenzung zu ersten Testversuchen während der Einschränkungsminderung, die ohne Unterbrechung des laufenden Betriebs durchgeführt werden. Es werden Lösungsansätze zur Minderung der dabei aufgedeckten Einschränkungen erarbeitet, was zu zusätzlichen konzeptionellen Anpassungen führen kann. Im Gegensatz dazu werden die Anwendungsfälle und theoretisch erarbeiteten Lösungsansätze zur Einschränkungsminderung während der Testung erstmals im laufenden Betrieb oder mit externen Stakeholdern erprobt. Damit stellt die Testung eine abschließende Phase dar, deren positiver Ausgang für Entscheidungen im Hinblick auf die Frage der Fortführung des Projekts ausschlaggebend ist.

Läuft der Testlauf nicht wie erhofft, ließen sich zwei Wege einschlagen: Zum einen könnte dies zur erneuten Betrachtung der Anwendungsfälle führen und dafür sorgen, dass sie neu definiert werden. Zusätzlich könnte die Testung weitere Einschränkungen aufdecken, die vor der Implementierung eine Minderung erfahren sollten. Es besteht somit eine wechselseitige Beziehung zwischen der Testung und den Aktivitäten der konzeptionellen Anpassung und Einschränkungsminderung. Wichtig ist, dass aus Sicht der Unternehmensführung auch bei erneutem Durchlaufen dieser Aktivitäten erst eine erfolgreiche Testung das Ende der Experimentierphase bildet. Ein negatives Ergebnis kann zum Abbruch des Projekts führen, weil z. B. kein Mehrwert gesehen wird.

**Abb. 1:**
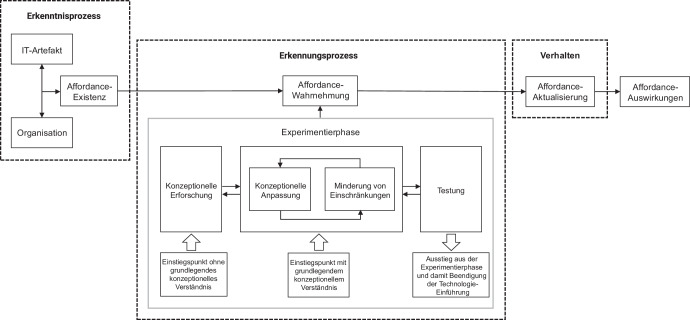
Erweitertes Affordance-Prozess-Modell (adaptiert nach Pozzi et al. 2014)

Zusammenfassend lässt sich die Existenz der Experimentierphase bekräftigen. Die bestehenden Aktivitäten konnten unter Verwendung von VR in einem Unternehmen ermittelt und um eine zusätzliche Aktivität erweitert werden. Es stellt sich jedoch noch die Frage, wo die Experimentierphase im Prozess-Modell nach Pozzi et al. (2014) einzuordnen ist. Die Ergebnisse dieser Studie deuten darauf hin, dass die Experimentierphase besonders bei neuartigen Technologien die Wahrnehmung von Affordances unterstützt. Es bietet sich also an, das Modell gemäß Abb. 1 zu erweitern.

### Theoretischer Mehrwert und praktische Implikationen

Zum einen unterstützen die Ergebnisse die Annahmen der A‑E-A-Theorie (Du et al. 2019). Es ist davon auszugehen, dass die Erweiterung der Experimentierphase um die Testung auf die Einführung anderer innovativer Technologien übertragen werden kann. Hierbei ist anzumerken, dass insbesondere bei hohem Vorbekanntheitsgrad einer Technologie einzelne Aktivitäten auch stärker ineinander übergehen und bspw. erste Überlegungen hinsichtlich der Einschränkungsminderung schon während der konzeptionellen Anpassung angestellt werden. Außerdem wird die Experimentierphase in ein bestehendes Prozess-Modell eingeordnet und damit von anderen Phasen wie der Wahrnehmung oder Aktualisierung deutlicher abgegrenzt (Pozzi et al. 2014).

Für die Praxis dienen die identifizierten Aktionspotenziale als Ansatzpunkte für VR-Einsatzmöglichkeiten in vergleichbaren Unternehmen. Zusätzlich zeigen die Ergebnisse, wie Unternehmen eine erfolgreiche VR-Experimentierphase gestalten können. Zu Beginn der Experimentierphase können Schulungen von Softwareanbietern dabei unterstützen, ein konzeptuelles Grundverständnis zu erlangen. Des Weiteren wird eine gesonderte Umgebung wie eine Zukunftswerkstatt empfohlen, um ohne Störung des laufenden Betriebs neue Software und Hardware zu erforschen. Es hat sich auch gezeigt, dass eine Offenheit gegenüber VR-Erfahrungen aus anderen Bereichen (z. B. Spielebranche) nützlich sein kann, da diese als Inspiration für die Entwicklung von sinnvollen Anwendungsfällen dienen können. Vor der Implementierung sollten zudem mögliche Einschränkungen identifiziert und Lösungsvorschläge erarbeitet werden. Bei PureAir wurde bspw. die Anschaffung von mobilen VR-Sets empfohlen, um unhandliche Produkte virtuell bei Kunden „vor Ort“ präsentieren zu können. Sollte sich VR weiter in der Unternehmenswelt verbreiten, muss jedoch über standardisierte Schnittstellen für unterschiedliche VR-Hardware bzw. -Software nachgedacht werden. Des Weiteren wurde betont, dass VR nicht eingesetzt werden sollte, um Kollaboration und Marketing mit Geschäftspartnern und Kunden vollständig zu virtualisieren, sondern um ein Erlebnis zu schaffen. So könnten Beschäftigte im Vertrieb nach wie vor zu Kunden fahren und mit einer VR-Produktpräsentation eine einzigartige Erfahrung bieten. Die identifizierten Anwendungsfälle, Einschränkungen und Lösungsvorschläge sollten in einem Testlauf unter realen Bedingungen erprobt werden, um den Mehrwert und die Umsetzbarkeit für die Unternehmensführung aufzuzeigen.

### Limitationen und zukünftige Forschung

Eine grundsätzliche Limitation ist die Generalisierbarkeit von Einzelfallstudien, diese sind jedoch geeignet, wenn es sich um einen einzigartigen und aufschlussreichen Fall handelt. Diese Bedingungen waren bei PureAir gegeben, da das Unternehmen die seltene Möglichkeit bot, die Experimentierphase in Bezug auf VR zu untersuchen. Darüber hinaus wurde ein ähnliches Studiendesign wie bei bisheriger Forschung gewählt, um Ergebnisse vergleichen zu können. Des Weiteren handelt es sich bei dem Einzelfall um ein internationales Unternehmen, das in der Entwicklung, Produktion und dem Vertrieb tätig ist, weswegen die Ergebnisse möglicherweise nicht auf Betriebe abweichender Größe oder Branche übertragbar sind.

Zukünftige Forschung könnte die Experimentierphase in Bezug auf weitere innovative Technologien und in anderen Branchen untersuchen, um weitere Unterschiede bezüglich der erfolgreichen Vorbereitung auf die Implementierung aufzudecken. Darüber hinaus könnte eine Abstraktion der erzielten Ergebnisse hilfreich sein, um einen Methoden‑/Toolkoffer für den VR-Einsatz in bestimmten Anwendungsfällen zu entwickeln. Ein solcher Methoden‑/Toolkoffer könnte genutzt werden, um die Umsetzbarkeit von Anwendungsfällen mit entsprechenden VR-Lösungen einzuordnen und Investitionsentscheidungen zu unterstützen. Interessant wäre auch ein Vergleich der Experimentierphase mit ähnlichen Phasenkonzepten der Prozessverbesserung wie bspw. dem PDCA-Zyklus aus dem Lean Management.
